# Placenta Percreta Invading Broad Ligament and Parametrium in a Woman with Two Previous Cesarean Sections: A Case Report

**DOI:** 10.1155/2012/251381

**Published:** 2012-10-14

**Authors:** Mansoureh Vahdat, Abolfazl Mehdizadeh, Elaheh Sariri, Shahla Chaichian, Zahra Najmi, Maryam Kadivar

**Affiliations:** ^1^Department of Obstetrics and Gynecology, Minimally Invasive Surgery Research Center, Rasool-e-Akram Hospital, Tehran University of Medical Sciences, Tehran, Iran; ^2^Department of Pathology, Rasool-e-Akram Hospital, Tehran University of Medical Sciences, Tehran, Iran

## Abstract

*Introduction*. The incidence of placenta accreta has dramatically increased due to increasing caesarean section rate all over the world. Placenta percreta is the most severe form of placenta accretes. It frequently results in maternal morbidity and mortality mainly caused by massive obstetric hemorrhage or emergency hysterectomy. Percreta invading into the broad ligament has rarely been previously reported. *Case presenting*. We presented a case of placenta percreta invading left broad ligament and parametrium in a woman with two previous cesarean sections, which led to massive intraoperative hemorrhage during hysterectomy and transient ischemic encephalopathy. *Conclusion*. In cases of parametrial involvement, it would be more difficult to decide whether to remove placenta or leave it in site. In surgical removal neither local excision of placental bed and uterine repair nor traditional hysterectomy is adequate if parametrium invaded by placenta. We suggest delayed elective hysterectomy in such cases. So, pregnancy-induced pelvic congestion would be decreased, we can gather an expert team of gynecologists, urologists, and vascular surgeons, we could get plenty of blood products, and we may have the chance to administer methotrexate.

## 1. Introduction

Placenta accreta is an anomaly in placentation leading to its abnormal firm attachment to the myometrium, due to the absence of decidua basalis and leads to its incomplete separation at the time of delivery [1]. Superficial, middle layer, and deep placenta invasion are termed as placenta accreta, increta, and percreta, respectively; however, all three types are collectively known as placenta accreta [2].

Due to increasing caesarean section rate all over the world (from 5 to 8% to 25–30%), the incidence of placenta accreta has dramatically increased [2]—nearly 10-fold in the past 50 years [3]. Percreta is the most severe form of placenta accretes, which comprises 5% to 7% of these cases [3]. It frequently results in maternal morbidity and mortality being as high as 7%, mainly caused by massive obstetric hemorrhage or emergency hysterectomy as well as increased surgical risks to the surrounding structures [1–3].

Placenta percreta may invade the rectum, bladder, and even parametrium. Invasion to the broad ligament has rarely been previously reported [4, 5]. Here we present a case of placenta percreta invading left broad ligament and parametrium in a woman with two previous cesarean sections with massive intraoperative hemorrhage.

## 2. Case Presentation

**Figure 1:**
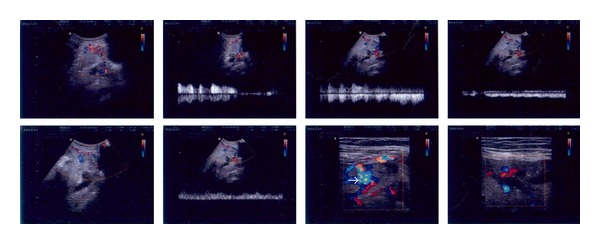
Ultrasound feature of percreta, in 37th week of gestation. Arrow shows lacunas in placenta.

A 35-year-old woman, gravid 4, para 2, live 2, with one abortion and two previous transverse lower-segment cesarean deliveries, was referred for labor pain at 38 weeks' gestation. Her ultrasound findings were consistent with placenta previa and accrete, based on the presence of various lacunas in placenta with pathological “storm flow” on Doppler ultrasound (Figure 1).

On primary examination, she was hemodynamically stable, but forceful uterine contractions were noted. Fetus was breech in leopold examination. She had no complaint of vaginal bleeding or ruptures of membrane, and as for the placenta previa reported in her ultrasound reports, we did not perform vaginal examination. An emergency caesarean section was performed under general anesthesia with midline abdominal and classic uterine incision. Anterior wall of lower segment of the uterus was full of dilated veins, and placenta was visible in the left broad ligament.

**Figure 2:**
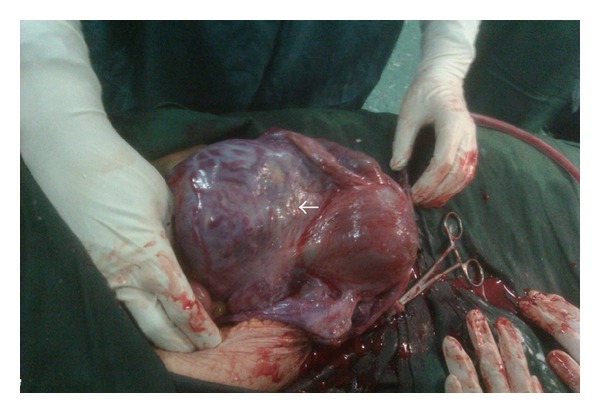
Placenta invading left broad ligament and parametrium. Arrow is pointed to the placenta.

Uterine classic incision was made on the fondus, and the fetus was delivered as with cephalic delivery. Fortunately, the placenta did not injure during incision and delivery. By more exploration, placenta percreta, originating from postero-lateral wall of the uterus, and forming a mass in douglas cavity, was clinically diagnosed. Placenta also invaded towards left parametrium, involving left infundibulopelvic ligament, too (Figure 2).

Placental mass was covered with a thin serosa and was prone to rupture and bleeding even with mild trauma. So we decided to perform hysterectomy. First, bilateral internal iliac artery ligation was performed, and patient underwent supracervical hysterectomy. However, it did not prevent the massive bleeding from the remaining uterus and the remaining placenta on parametrium, douglas cavity, and posterior wall of the bladder. The remaining part of the cervix was extracted, and radical parametrectomy was also necessary to remove the mass.

However bleeding did not stop and despite meticulous ligation of all visible bleeders, diffuse oozing continued. As patient became hemodynamically unstable, we decided to finish the surgical procedure after packing the abdominal cavity with longaxes and leaving a drain. Four days later in a second-look surgery longaxes were removed.

An estimated blood loss of 7000 mL occurred during operation. Patient had one episode of cardiac arrest during the operation which resuscitated. She was transfused with 20 units of packed red blood cells, 15 units of FFP, and 15 units of platelet during operation and in postoperative period.

She was intubated for 10 days and managed conservatively during this period in intensive care unit (ICU). Her brain imaging showed hypoxic ischemic encephalopathy. She experienced a transient ischemic encephalopathy, which improved gradually from GCS 6 in first postoperative day to 12 when we succeed to extubate her and 15 when she discharged from ICU. Finally she discharged from hospital on 30th postoperative day with some motor sequels in her lower extremities, needing physiotherapy and exercise.

**Pathologic Findings**

**Figure 3:**
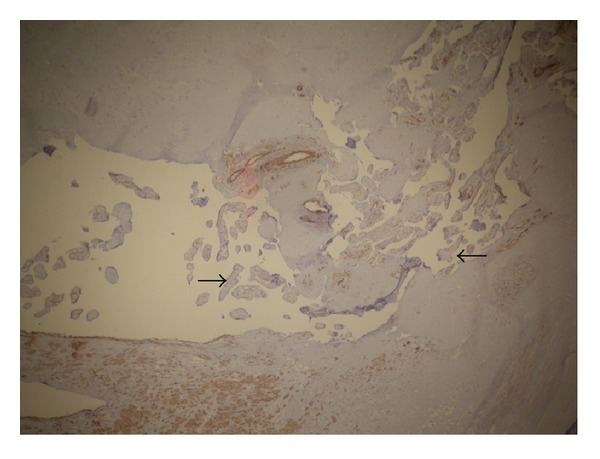
Pathologic feature shows placenta increta.

Macroscopically, more than 70% of the placenta was extrauterine, which diagnosed as placenta percreta. In histological examination, the myometrium was intact all over the uterus, with no evidence of villous invasion except for the site of previous kerr incision scar, compatible with the diagnosis of placenta increta (Figure 3). Surprisingly, the pathology report did not confirm invasion to the serosa, percreta.

Why the histological exam could not confirm the macroscopic diagnosis is a question. We assume that this case may be some kind of cesarean scar implantation of the placenta, which invaded and reached extrauterine space early in its development. So the placenta could grow in a high vascular bed of the broad ligament, and on the other hand as the invasion occurred very soon in placental development, the small part of invasion in histological examination was limited to the site of previous uterine scar. 

## 3. Discussion

Two types of managements for placenta percreta have been proposed: (1) surgical removal of the uterus and the involved tissues, and (2) conservative management. 93% of obstetricians still prefer a radical surgical approach [6]; however, it carries a high maternal morbidity like urethral ligation (5%) and mortality (7%) [7]. One of the largest meta-analysis of 54 placentas percreta invading the bladder showed a high maternal mortality (6%) with 44% of patients undergoing partial or total cystectomy [8]. Hysterectomy has been the traditional treatment for placenta percreta; however, when placenta percreta invades neighboring organs, bleeding may be extreme and surgery is very hazardous. 

More recently, conservative management leaving the placenta, completely or partially, in place with or without administering methotrexate, has been advocated [9], and similarly, internal iliac arteries catheterization with occlusion or uterine artery embolization [10]. Conservative treatment is not without complications which may include catastrophic bleeding, vaginal necrosis, paresthesia of the lower extremities, and arterial thrombosis [11]. It causes four times higher mortality rate than the immediate surgical treatment. Moreover, this approach cannot be applied in emergency settings for unstable patients.

Invasion of parametrium is probably a rare event in cases of placenta percreta, and its management would be more challenging [4, 5]. In surgical removal, neither local excision of the placental bed and uterine repair nor traditional hysterectomy is adequate if the parametrium was invaded by placenta. We performed a left parametrectomy to stop severe bleeding and eventually we packed the abdomen. In our case, bilateral hypogastric ligation couldnot stop diffuse bleeding; it raises the question whether internal iliac arteries catheterization/embolization could help in situations of extensive parametrial invasion with diffuse collateral perfusions. It is not well clear yet, but it seems to be at least helpful to some extent, in reducing the amount of massive bleeding.

 Could we leave the placenta in situ and repair the uterine incision? Previous experiences shows it could be a successful management in some case of placenta accrete [12, 13], but some were complicated with infection [14] and massive bleeding [14, 15], and some of them finally underwent delayed hysterectomy [14, 15]. There is no previous report of conservative management in cases of broad ligament placenta. As the only support of the placenta is the peritoneal layer of broad ligament, which stretches more and more by growth of the placenta, the probability of the rupture, and massive intraabdominal bleeding is high in these cases, as reported in one case which led to acute abdominal pain [4].

However, we suggest delayed elective hysterectomy in such cases. To open the uterine with classic incision as high as it does not harm the placenta, to close the incision and observe patient in ICU for few days, and to perform hysterectomy 7–10 days later. In this period, pregnancy- induced pelvic congestion would be decreased, we can gather an expert team of gynecologists, urologists, and vascular surgeons, we could get plenty of blood products, and we may have the chance to administer methotrexate.

## 4. Conclusion

In cases of placenta percreta with parametrial involvement, it would be more difficult to decide whether to remove or leave the placenta. In surgical removal, neither local excision of placental bed and uterine repair nor traditional hysterectomy would be adequate. Leaving the placenta in situ has its own complications too. However delayed elective hysterectomy would let to gather an expert team. We may have the chance to administer methotrexate and to perform our surgery after decreasing the pregnancy-induced pelvic congestion.
